# Pregnancy-Related Hormones Increase UGT1A1-Mediated Labetalol Metabolism in Human Hepatocytes

**DOI:** 10.3389/fphar.2021.655320

**Published:** 2021-04-15

**Authors:** Raju Khatri, John K. Fallon, Craig Sykes, Natasha Kulick, Rebecca J. B. Rementer, Taryn A. Miner, Amanda P. Schauer, Angela D. M. Kashuba, Kim A. Boggess, Kim L. R. Brouwer, Philip C. Smith, Craig R. Lee

**Affiliations:** ^1^ Division of Pharmacotherapy and Experimental Therapeutics, UNC Eshelman School of Pharmacy, University of North Carolina at Chapel Hill, Chapel Hill, NC, United States; ^2^ Division of Pharmacoengineering and Molecular Pharmaceutics, UNC Eshelman School of Pharmacy, University of North Carolina at Chapel Hill, Chapel Hill, NC, United States; ^3^ Department of Obstetrics and Gynecology, UNC School of Medicine, University of North Carolina at Chapel Hill, Chapel Hill, NC, United States

**Keywords:** pregnancy, uridine diphosphate glucuronosyltransferases, hepatic metabolism, estradiol, progesterone, labetalol, hypertension, targeted proteomics

## Abstract

Pregnancy-related hormones (PRH) are recognized as important regulators of hepatic cytochrome P450 enzyme expression and function. However, the impact of PRH on the hepatic expression and function of uridine diphosphate glucuronosyltransferases (UGTs) remains unclear. Using primary human hepatocytes, we evaluated the effect of PRH exposure on mRNA levels and protein concentrations of UGT1A1, UGT2B7, and other key UGT enzymes, and on the metabolism of labetalol (a UGT1A1 and UGT2B7 substrate commonly prescribed to treat hypertensive disorders of pregnancy). Sandwich-cultured human hepatocytes (SCHH) from female donors were exposed to the PRH estradiol, estriol, estetrol, progesterone, and cortisol individually or in combination. We quantified protein concentrations of UGT1A1, UGT2B7, and four additional UGT1A isoforms in SCHH membrane fractions and evaluated the metabolism of labetalol to its glucuronide metabolites in SCHH. PRH exposure increased mRNA levels and protein concentrations of UGT1A1 and UGT1A4 in SCHH. PRH exposure also significantly increased labetalol metabolism to its UGT1A1-derived glucuronide metabolite in a concentration-dependent manner, which positively correlated with PRH-induced changes in UGT1A1 protein concentrations. In contrast, PRH did not alter UGT2B7 mRNA levels or protein concentrations in SCHH, and formation of the UGT2B7-derived labetalol glucuronide metabolite was decreased following PRH exposure. Our findings demonstrate that PRH alter expression and function of UGT proteins in an isoform-specific manner and increase UGT1A1-mediated labetalol metabolism in human hepatocytes by inducing UGT1A1 protein concentrations. These results provide mechanistic insight into the increases in labetalol clearance observed in pregnant individuals.

## Introduction

Approximately 80% of pregnant individuals take at least one medication for the treatment of either a preexisting chronic or acute condition during pregnancy (Ayad and Costantine, 2015). Physiological and biochemical changes of pregnancy can change the pharmacokinetics and effects of certain drugs; however, most drugs used in pregnancy do not have dosing information specific to these patients (Gonzalez et al., 2015; Tasnif et al., 2016; Dallmann et al., 2018a). More precise dosing recommendations in pregnant individuals are lacking, in part, due to a poor understanding of key factors that alter hepatic drug disposition during pregnancy.

Numerous pregnancy related hormones (PRH), including cortisol (CRT), progesterone (P4), and various estrogens increase substantially during pregnancy (Soldin et al., 2005; Holinka et al., 2008; Choi et al., 2013; Papageorgiou et al., 2013). Accumulating evidence has demonstrated that PRH significantly alter hepatic mRNA levels and metabolic activity of certain cytochrome P450 enzymes, most notably CYP2B6 and CYP3A4 (Choi et al., 2013; Papageorgiou et al., 2013; Zhang et al., 2015; Khatri et al., 2021), which has been hypothesized as a central mechanism underlying altered hepatic clearance of cytochrome P450 substrates during pregnancy *in vivo* (Jeong, 2010; Isoherranen and Thummel, 2013; Dallmann et al., 2018b). In contrast, the impact of PRH on hepatic expression and function of phase II drug metabolizing enzymes (DMEs) has not been investigated as rigorously. In particular, significant gaps in knowledge remain surrounding the mechanisms underlying pregnancy-associated changes in the expression and function of uridine diphosphate glucuronosyltransferases (UGTs), a key family of phase II DMEs involved in the conjugation and clearance of numerous drug substrates (Williams et al., 2004; Anderson, 2005; Fujiwara et al., 2016).

Estradiol (E2) and P4 increase *UGT1A1* mRNA in hepatocytes isolated from humanized UGT1 (hUGT1) mice. Compared to non-pregnant controls, pregnant mice exhibit higher liver expression of UGT1A1, UGT1A4, and other UGT1A isoforms by activating pregnane X receptor (PXR)- and constitutive androstane receptor (CAR)-dependent transcription (Chen et al., 2012; Liao et al., 2018). In HepG2 cells, P4 increases *UGT1A1* promoter activity via PXR activation and E2 increases *UGT1A4* promoter activity via estrogen receptor alpha (ERα); in contrast, neither E2 nor P4 alters *UGT2B7* promoter activation (Jeong et al., 2008; Chen et al., 2009). Although these studies provide insight into the molecular underpinnings of UGT regulation by PRH, it remains unknown whether PRH alter UGT mRNA and protein expression and UGT-mediated glucuronidation of clinically relevant drugs commonly prescribed during pregnancy in human hepatocytes.

Hypertensive disorders of pregnancy are among the most common chronic medical conditions encountered in pregnancy (Townsend et al., 2016; Webster et al., 2017a). Hypertension is a major risk factor for maternal and fetal morbidity and mortality, and high blood pressure is associated with increased risk of adverse pregnancy outcomes; thus, antihypertensive drugs are commonly prescribed to lower maternal blood pressure and promote maternal and fetal health (Seely and Ecker, 2011; Webster et al., 2017a; Cleary et al., 2018). Labetalol is the first-line agent for hypertension treatment in pregnancy and commonly prescribed to pregnant individuals (Clark et al., 2015; Magee et al., 2015; Webster et al., 2017b; Cleary et al., 2018). Labetalol, a UGT1A1 and UGT2B7 substrate (Jeong et al., 2008), is extensively metabolized in the liver to glucuronide metabolites and less than 5% of the dose is excreted unchanged in the urine (Martin et al., 1976; Yeleswaram et al., 1993). Oral labetalol is absorbed rapidly, but undergoes extensive first-pass metabolism resulting in an average oral bioavailability of 18–35% (Kirsten et al., 1998). Approximately 50–60% of labetalol is bound to plasma proteins, and its plasma elimination half-life is approximately 8 h in non-pregnant individuals (Kirsten et al., 1998; Clark et al., 2015). Labetalol clearance increases during pregnancy, leading to a shorter elimination half-life, frequent treatment failures, and the need for higher doses to control blood pressure (Rogers et al., 1990; Saotome et al., 1993; Kirsten et al., 1998; Hardman et al., 2005; Fischer et al., 2014; Clark et al., 2015). A population pharmacokinetic analysis suggested that gestational age-dependent increases in labetalol oral clearance observed during pregnancy were most likely mediated by an increase in hepatic intrinsic clearance (Fischer et al., 2014). However, despite the reported effects of PRH on UGT regulation (Jeong et al., 2008; Chen et al., 2012), the impact of PRH on UGT1A1 and UGT2B7 expression and labetalol metabolism in human hepatocytes has not been studied.

The primary objectives of the current investigation were to evaluate the effects of PRH on (1) the protein concentrations of UGT1A1, UGT2B7, and other key UGT1A enzymes, and (2) UGT1A1-and UGT2B7-mediated glucuronidation of labetalol in sandwich-cultured human hepatocytes (SCHH).

## Materials and Methods

### Chemicals and Reagents

All reagents were obtained from ThermoFisher Scientific (Waltham, MA) unless otherwise indicated. Dimethyl sulfoxide (DMSO), labetalol, 17β-estradiol (E2), estriol (E3), estetrol (E4), progesterone (P4), cortisol (CRT), rifampin, and 6-(4-Chlorophenyl) Imidazo [2,1-b][1,3] Thiazole-5-Carbaldehyde o-(3,4-dichlorobenzyl) Oxime (CITCO) were purchased from Sigma-Aldrich (St. Louis, MO). Matrigel® matrix and Biocoat™ Collagen I Coated Multiwell Plates were obtained from Corning (Bedford, MA). Labetalol-d_3_ was purchased from Toronto Research Chemicals (Toronto, ON, Canada).

### Sandwich-Cultured Human Hepatocytes

Primary human hepatocytes were purchased in cryopreserved vials from Life Technologies (Carlsbad, CA) and Xenotech (Kansas City, KS). Hepatocytes from female donors ages 18–49 years (reproductive age range defined by the World Health Organization) were used in the current experiments. The donor characteristics are summarized in (Supplementary Table S1). Hepatocytes were plated and cultured as SCHH at 37°C in 5% CO_2_ using previously described methods (Swift et al., 2010; Khatri et al., 2021). Briefly, hepatocytes were thawed in Hepatocyte Thaw Medium (Life Technologies, Carlsbad, CA). Thawed hepatocytes were then plated in QualGro™ Seeding Medium (BioIVT, Durham, NC) on Corning Biocoat™ Collagen I Multiwell Plates at the following densities: 0.5 million cells per well in 24-well plates for RNA and metabolism experiments; 1 million cells per well in 12-well plates for quantitative proteomics experiments, as previously described (Khatri et al., 2021). The following day (day 1), SCHH were established by overlaying the plated hepatocytes with Corning Matrigel® Matrix in QualGro™ Cell Culture Medium (BioIVT, Durham, NC). The following day (day 2), the SCHH were simultaneously incubated in QualGro™ Induction Medium (BioIVT, Durham, NC) and treated with either PRH, vehicle control (0.1% DMSO), or established activators (positive controls) of PXR (rifampin, 10 μM) and CAR (CITCO, 1 μM) for 72 h.

In each experiment, as previously described (Khatri et al., 2021), E2, E3, E4, P4, and CRT were combined and exogenously administered to SCHH as a PRH cocktail (CKTL) to simulate exposure to increased concentrations of multiple hormones during pregnancy. In parallel, individual hormones were administered to SCHH in order to distinguish the effects of each PRH relative to the CKTL and controls. It is well-established that PRH exhibit a range of plasma concentrations *in vivo*; notably, circulating concentrations of total P4 and CRT are approximately 10-fold higher than E2 (Tulchinsky et al., 1972; Soldin et al., 2005; Papageorgiou et al., 2013; Zhang et al., 2015). In addition, human hepatocytes are known to rapidly metabolize E2 and P4 *in vitro* (half-life of approximately 1–2 h), while CRT undergoes minimal metabolism in human hepatocytes (Choi et al., 2013; Zhang et al., 2015). Consequently, exogenous administration of approximately 1 μM of E2, 1 μM of CRT, and 10 μM of P4, with repeated administration every 6–12 h throughout the 72 h treatment period, has been reported to yield average hormone concentrations in cultured hepatocytes that approximate total plasma concentrations during the third trimester (T3) *in vivo* (Koh et al., 2012; Papageorgiou et al., 2013; Zhang et al., 2015). Accordingly, the individual PRH and the PRH CKTL were exogenously administered to SCHH at two concentrations (1 µM or 10 μM) to evaluate concentration-dependent effects. In order to sustain the desired average PRH concentrations in SCHH throughout the 72 h induction period, the media with PRH was replaced at 8, 24, 32, 48, and 56 h. At 72 h, SCHH were washed and incubated with labetalol for metabolism experiments, or harvested for isolation of either mRNA or membrane-associated protein.

### RNA Isolation and Quantitative PCR

Total RNA was isolated from SCHH (donors HU1880, HC3-26, HU8284, and HC3-40) using the RNeasy Miniprep Kit (Qiagen, Valencia, CA). Total RNA was then reverse transcribed to cDNA using a High Capacity cDNA Reverse Transcription Kit (Applied Biosystems, Foster City, CA) according to the manufacturer’s instructions. mRNA levels of key UGT isoforms were quantified by quantitative real-time PCR using the Applied Biosystems QuantStudio(™) 6 Flex System (Applied Biosystems, Foster City, CA) and Taqman® gene expression assays (*UGT1A1*: Hs02511055_s1, *UGT1A3:* Hs04194492_g1, *UGT1A4:* Hs01655285_s1, *UGT1A6:* Hs01592477_m1, *UGT1A9:* Hs02516855_sH, *UGT2B7*: Hs00426592_m1, and *GAPDH*: Hs02758991_g1). As previously described (Khatri et al., 2021), each reaction was performed in duplicate with 10 ng of cDNA and 2x Taqman universal PCR Master Mix (Applied Biosystems, Foster City, CA), and UGT mRNA levels were normalized to *GAPDH* and expressed relative to the DMSO control group using the 2^−ΔΔCt^ method.

### Membrane-Associated Protein Isolation and Quantitative Targeted Absolute Proteomics

Membrane-associated protein was isolated from SCHH (donors HU1880, HC3-26, and HU8284) using the ProteoExtract® Native Membrane Protein Extraction Kit (EMD Millipore, Billerica, MA) with a differential buffer (surfactant) extraction procedure. As previously described (Khatri et al., 2019), hepatocytes (1 million cells/well) were incubated in Extraction Buffer I (750 µL) with gentle shaking at 4°C for 10 min, and centrifuged at 16,000 x g for 15 min to fractionate cytosolic proteins. The resulting cell pellets were suspended in Extraction Buffer II (100 µL), frozen at –80°C for 30 min, and incubated on ice for 15 min with gentle vortexing every 5 min, and then centrifuged at 16,000 x g for 15 min at 4°C. Membrane-associated protein in the supernatant was quantified using the Bio-Rad Protein Assay Kit II (Hercules, CA) and stored at −80°C.

From each sample, membrane-associated protein (30 μg) from was trypsin digested for 16 h, following denaturation, reduction and alkylation, as previously described (Fallon et al., 2013; Khatri et al., 2019). Sample clean-up was performed using solid phase extraction. Analysis of the resulting peptides (0.12 µg equivalent) for UGT1A1, UGT2B7, and four additional UGT1A isoforms (UGT1A3, UGT1A4, UGT1A6, and UGT1A9) was performed via nanoscale liquid chromatography coupled to tandem mass spectrometry (nanoLC-MS/MS) on a nanoAcquity (Waters, Milford, MA) coupled to a SCIEX QTRAP 5500 hybrid mass spectrometer (Framingham, MA) equipped with a NanoSpray III source, as previously described (Khatri et al., 2019). Briefly, peptides were eluted from the trap column and separated on a BEH130 C18 column (150 μm × 100 mm, 1.7 µm particle size; Waters, Milford, MA) at a flow rate of 1.3 μL/min with 1% acetonitrile in 0.1% formic acid and 100% acetonitrile under gradient conditions. The tandem mass spectrometry was conducted with ion spray voltage at 4000 in the positive mode. Heavy labeled peptide standards were used to quantify UGT protein concentrations, as previously described (Fallon et al., 2013; Khatri et al., 2019). The heavy labeled peptides used to report concentrations of each of the six UGT isoforms, and the MRMs acquired for each peptide, are shown in (Supplementary Table S2).

### Labetalol Metabolism

SCHH (donors HC3-26 and HU1880) were washed with William’s E Medium twice following the 72 h PRH induction period, and then incubated with William’s E Medium containing an enzyme-saturating concentration of labetalol (1 mM) for 4 h at 37°C (Jeong et al., 2008). Co-administration experiments with itraconazole (5 μM), a UGT1A1 inhibitor (Walsky et al., 2012), were performed to evaluate the contribution of UGT1A1 to PRH and rifampin-induced changes in labetalol metabolism. After the 4 h metabolism incubation period, SCHH lysates in 70% methanol and SCHH media were stored at −80°C prior to quantification of labetalol glucuronide formation by liquid chromatography tandem-mass spectrometry (LC-MS/MS). Due to the unknown contribution of drug transporters to labetalol disposition in hepatocytes, glucuronide formation was measured separately in SCHH cell lysates and media.

In addition, labetalol glucuronide formation was evaluated in recombinant UGT1A1 and UGT2B7 enzymes, as described (Wen et al., 2007). Briefly, labetalol (1 mM) was incubated with 0.5 mg/mL of human UGT1A1 or UGT2B7 Supersomes™ (Corning, cat. #456411 or 456427, respectively [Corning, NY]) in 100 µL reaction mixture containing 5 mM MgCl_2_ in freshly prepared 100 mM Tris buffer (pH 7.7). Reactions were incubated at 37°C for 5 min in a Fisher Scientific Isotemp Thermomixer (Hampton, NH). Then, freshly prepared UDPGA (co-factor, 5 mM final concentration) was added, quickly mixed by vortex for 2–3 s, and incubated in the Thermomixer with continuous shaking (100 rpm) at 37°C for 3 h. Cold methanol (235 µL) was added to stop labetalol metabolism and maintain 70% methanol (335 µL total volume). A follow-up co-administration experiment with the PRH CKTL (1 µM) was completed to determine whether labetalol glucuronide formation was altered by the presence of the PRH CKTL. Samples were stored at –80°C until measurement of labetalol glucuronide formation by LC-MS/MS.

### Measurement of Labetalol Glucuronide Formation by LC-MS/MS

Three glucuronide metabolites of labetalol have been detected (Martin et al., 1976; Niemeijer et al., 1991). Glucuronidation at the phenolic-OH (Gluc-1) by UGT1A1 and at the benzylic-OH (Gluc-2) by UGT2B7 have been previously reported (Jeong et al., 2008); however, it has not been reported which UGT isoform catalyzes the N-glucuronidation of labetalol (Gluc-3).

Media samples, cell lysate or recombinant reaction mixture (30 µL) were extracted by protein precipitation with methanol (150 µL) containing the stable, isotopically labeled internal standard labetalol-d_3_. Samples were vortexed and centrifuged, and 20 µL of the supernatant was mixed with 80 µL of water prior to LC-MS/MS analysis. Chromatographic separation of the three glucuronide metabolites was achieved on a Waters Atlantis T3 (50 × 2.1 mm, 3 µm particle size) analytical column with 0.1% formic acid in water and acetonitrile under gradient conditions. Analytes and internal standards were detected on SCIEX API 5000 triple quadrupole mass spectrometer using TurboIonSpray in the positive ionization mode. Due to the unavailability of analytical standards for labetalol glucuronides, the levels of the three glucuronides were assessed by the peak areas of each labetalol glucuronide (Gluc-1, Gluc-2, and Gluc-3) normalized to the peak area of internal analytical standard.

### Data Analysis

Data are presented as mean ± SEM and expressed relative to the DMSO (vehicle) control group, unless otherwise indicated. Expression and metabolism data were not normally distributed, and log-transformed prior to statistical analyses. In the SCHH experiments, data analysis was first conducted within each hepatocyte donor. The corresponding average value within each hepatocyte donor was then carried forward, when applicable, into analyses that combined data across donors. The effects of the PRH CKTL and individual PRH on UGT mRNA levels, UGT protein concentrations, and labetalol glucuronide metabolite formation were evaluated using a one-way ANOVA, followed by a post-hoc Fisher’s LSD test to compare differences across groups as previously described (Khatri et al., 2021). Pearson correlations were completed to evaluate the relationship between UGT mRNA levels, UGT protein concentrations, and labetalol glucuronide formation. In the recombinant enzyme experiments, labetalol glucuronide formation was expressed as a percentage relative to the highest glucuronide peak area. Data analysis were performed using GraphPad Prism 8.3 (GraphPad Software, La Jolla, CA) or SAS-JMP Pro 14 (SAS Institute, Cary, NC). For each analysis, a *p*-value of <0.05 was considered statistically significant.

## Results

### Pregnancy-Related Hormones Increase UGT mRNA Levels in SCHH in an Isoform-Specific Manner

**FIGURE 1:**
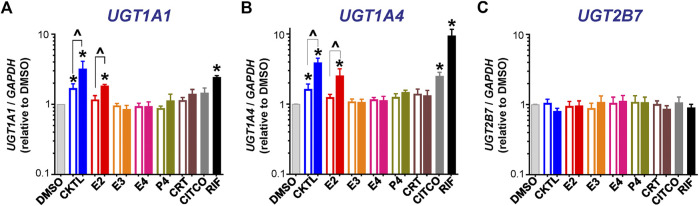
Effect of pregnancy-related hormones (PRH) on mRNA levels of key UGT isoforms in SCHH. Hepatocytes from four donors (HU1880, HC3-26, HU8284, and HC5-40) were exposed to E2, E3, E4, P4, and CRT, individually or in combination as a PRH cocktail [CKTL], or controls (DMSO, CITCO, Rifampin [RIF]) for 72 h (*n* = 2 per group within each hepatocyte donor). **(A)** *UGT1A1*, **(B)** *UGT1A4*, and **(C)** *UGT2B7* mRNA levels were normalized to *GAPDH* and expressed relative to the vehicle control group (DMSO) within each donor. The data were combined for comparison across experimental groups (*n* = 4 donors per group; mean ± SEM; *p < 0.05 vs. DMSO). Concentration-dependent effects were assessed (open bar: 1 μM, solid bar: 10 μM; ^*p* < 0.05 1 vs. 10 µM).

The PRH CKTL significantly increased *UGT1A1* mRNA levels (Figure 1A). The observed increase was concentration-dependent, driven by E2, and mirrored the induction effects of the PXR activator rifampin. The PRH CKTL and E2 also significantly increased *UGT1A4* mRNA levels in a concentration-dependent manner (Figure 1B). In contrast, *UGT2B7* mRNA levels were not altered by PRH in SCHH (Figure 1C). Evaluation of additional UGT1A isoforms revealed that *UGT1A3* mRNA levels were modestly induced by the PRH CKTL, and *UGT1A6* and *UGT1A9* mRNA levels were not altered by PRH (Supplementary Figure S1).

### Pregnancy-Related Hormones Increase UGT1A1, but not UGT2B7, Protein Concentrations in SCHH

**FIGURE 2:**
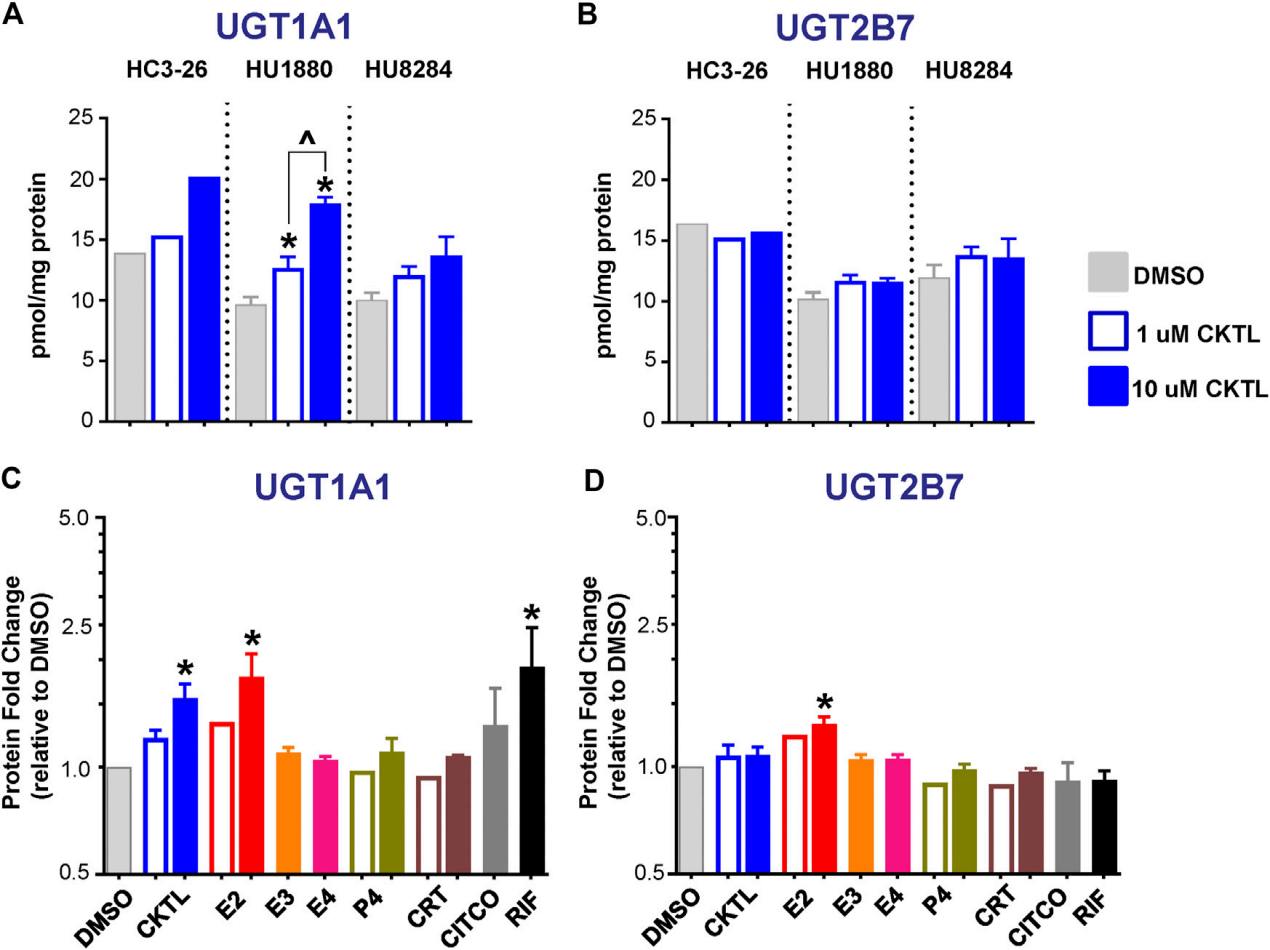
Effect of pregnancy-related hormones (PRH) on protein concentrations of UGT1A1 and UGT2B7 in SCHH. Following 72 h of hormone exposure, UGT1A1 and UGT2B7 protein concentrations were quantified by quantitative targeted absolute proteomics in SCHH membrane-associated protein fractions isolated from three donors (HC3-26, HU1880, and HU8284). **(A)** UGT1A1 and **(B)** UGT2B7 absolute protein concentrations in the DMSO and PRH cocktail (CKTL) groups were compared separately in donor HC3-26 (mean: *n* = 2 per group) and donors HU1880 and HU8284 (mean ± SEM: *n* = 3–4 per group; *p < 0.05 vs. DMSO, ^*p* < 0.05 1 vs. 10 µM). **(C)** UGT1A1 and **(D)** UGT2B7 protein concentrations were expressed relative to vehicle control (DMSO) within each hepatocyte donor, and then combined for comparison across groups. Open bars represent 1 µM CKTL (mean ± SEM: *n* = 3 donors per group) and 1 µM for the individual hormones (mean: *n* = 2 donors per group). Solid bars represent 10 µM CKTL and 10 µM for the individual PRH (mean ± SEM: *n* = 3 donors per group). *p < 0.05 vs. DMSO, ^*p* < 0.05 1 vs. 10 µM. Comparison of individual PRH effects within each hepatocyte donor are provided in Supplementary Figure S2.

The impact of PRH on UGT1A1 and UGT2B7 absolute protein concentrations were quantified and compared in SCHH membrane fractions from three female hepatocyte donors (Figure 2). The PRH CKTL appeared to increase UGT1A1 protein concentrations in each donor, with induction effects that were only observed at the high CKTL concentration in donor HC3-26, most pronounced and concentration-dependent in donor HU1880, and least pronounced in donor HU8284 (Figure 2A). In contrast, the PRH CKTL did not alter UGT2B7 protein concentrations in any of the three donors (Figure 2B).

Assessment of the average effect across hepatocyte donors demonstrated that the PRH CKTL significantly increased protein concentrations of UGT1A1 (Figure 2C), but not UGT2B7 (Figure 2D), compared to vehicle control. The PRH evoked changes in UGT1A1 protein concentrations exhibited a significant positive correlation with *UGT1A1* mRNA levels (r = 0.587, *p* < 0.001). UGT1A1 induction by the PRH CKTL appeared to be driven by E2, which also increased UGT1A1 protein concentrations in a concentration-dependent manner and mirrored the induction effects of rifampin on UGT1A1 across donors (Figure 2C) and within each donor (Supplementary Figure S2A). UGT1A1 protein concentrations were not increased by E3, E4, P4 or CRT. Although UGT2B7 protein concentrations were not significantly increased by the PRH CKTL, 10 μM E2 increased UGT2B7 compared to vehicle control (Figure 2D); the magnitude of this effect was smaller than observed with UGT1A1, but was apparent within each hepatocyte donor (Supplementary Figure S2B).

### Labetalol is Metabolized by UGT1A1 and UGT2B7 to Distinct Glucuronide Metabolites

**FIGURE 3:**
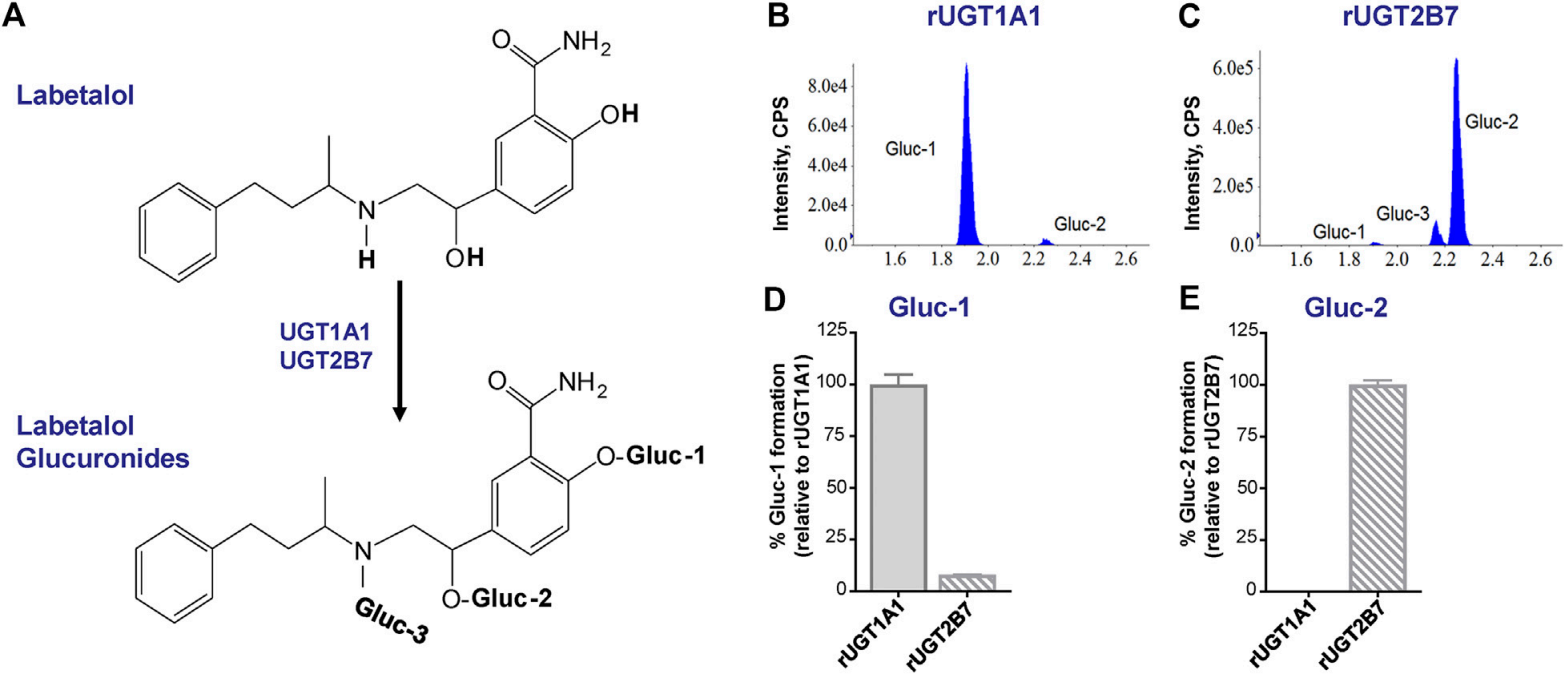
UGT1A1 and UGT2B7-mediated glucuronidation of labetalol. **(A)** Overview of labetalol glucuronidation to its phenolic-OH (Gluc-1), benzylic-OH (Gluc-2), and N-glucuronide (Gluc-3) metabolites. Representative chromatograms of labetalol glucuronide (Gluc-1, Gluc-2, and Gluc-3) formation by human recombinant **(B)** UGT1A1 and **(C)** UGT2B7. Relative formation of **(D)** Gluc-1 and **(E)** Gluc-2 by human recombinant UGT1A1 and UGT2B7. Data are expressed as a percentage of Gluc-1 formed by UGT1A1 and Gluc-2 formed by UGT2B7, respectively (*n* = 3/group; mean ± SEM).

Labetalol has three sites of glucuronidation (Figure 3A). Consistent with prior reports (Jeong et al., 2008), evaluation of glucuronide formation by human recombinant UGT1A1 and UGT2B7 enzymes demonstrated that glucuronidation of labetalol at the phenolic-OH position (Gluc-1) and at the benzylic-OH position (Gluc-2) was the primary glucuronide metabolite formed by UGT1A1 (Figure 3B) and UGT2B7 (Figure 3C), respectively. Gluc-1 was formed by both UGT1A1 and UGT2B7, however, Gluc-1 formation by UGT2B7 was minor compared to UGT1A1 (Figure 3D). Although detectable, Gluc-2 formation by UGT1A1 was negligible compared to UGT2B7 (Figure 3E). We also observed that UGT2B7, but not UGT1A1, catalyzed formation of the N-glucuronide (Gluc-3) metabolite as a minor product (Figure 3C).

### Effect of Pregnancy-Related Hormones on UGT1A1-Mediated Labetalol Glucuronidation in SCHH

**FIGURE 4:**
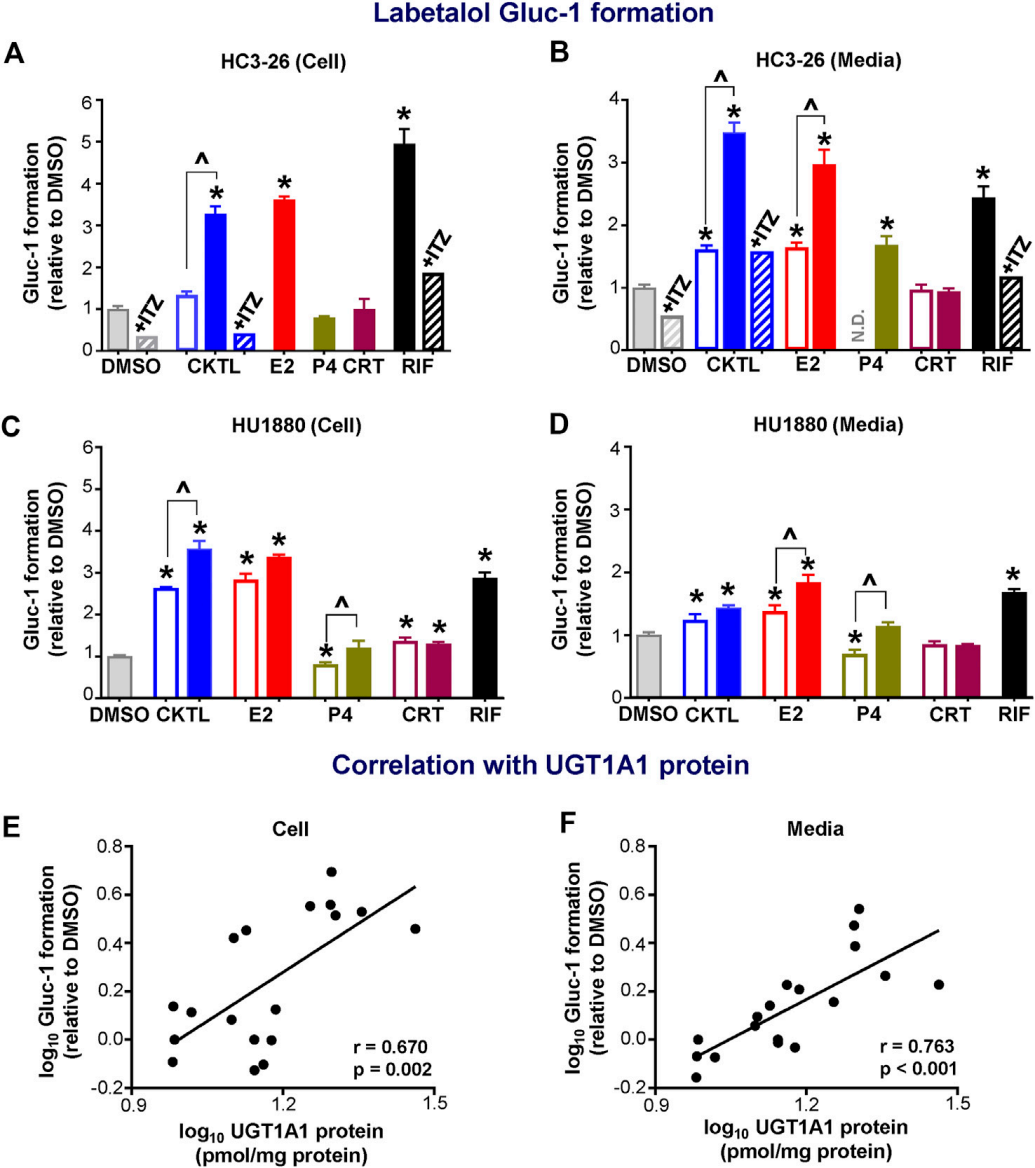
Effect of pregnancy-related hormones (PRH) on labetalol glucuronide (Gluc-1) formation in SCHH. Following 72 h of PRH exposure, SCHH from two donors (HC3-26, HU1880) were incubated with labetalol (1 mM) for 4 h. Labetalol glucuronide (Gluc-1) peak area was measured in both SCHH cell lysates **(A, C)** and SCHH media **(B, D)**, normalized to internal standard (labetalol-d_3_) peak area in each sample, and compared across treatment groups in donor HC3-26 (A: cell lysate, *n* = 3 per group; B: media, *n* = 3–6 per group) and donor HU1880 (C: cell lysate, *n* = 4 per group; D: media, *n* = 4 per group). In the donor HC3-26 experiment, cell lysates were not harvested for glucuronide measurements in the 1 µM E2, P4, or CRT groups. Data are calculated as the fold-change in Gluc-1 metabolite formation relative to vehicle control (*p < 0.05 vs. DMSO). Concentration-dependent effects were assessed (open bar: 1 μM, solid bar: 10 μM; ^*p* < 0.05 1 vs. 10 µM). In the donor HC3-26 experiments, itraconazole co-administration (+ITZ, 5 μM) was included in the DMSO-, PRH cocktail (CKTL)-, and rifampin (RIF)-treated groups (*n* = 1 per group in cell lysates; *n* = 2 per group in media) to confirm UGT1A1-mediated effects. N.D., experimental group not studied. The correlation between UGT1A1 protein levels and labetalol Gluc-1 formation in **(E)** SCHH cell lysates and **(F)** SCHH media in both donors is presented. Each data point represents the mean fold-change value for the various treatment groups, relative to DMSO, within each hepatocyte donor. The Pearson correlation coefficient (r) and *p*-value are provided.

Given the observed impact of PRH on UGT1A1 protein concentrations, we quantified the impact of PRH on labetalol Gluc-1 formation in SCHH. Rifampin significantly increased labetalol Gluc-1 formation in both hepatocyte donors (Figure 4). Compared to vehicle control, the PRH CKTL significantly increased labetalol Gluc-1 formation in a concentration-dependent manner in cell lysates from donor HC3-26 (1 μM: 1.33 ± 0.15-fold and 10 μM: 3.27 ± 0.31-fold, Figure 4A) and donor HU1880 (1 μM: 2.64 ± 0.04-fold and 10 μM: 3.57 ± 0.37-fold; Figure 4C). The PRH CKTL also significantly increased labetalol Gluc-1 formation in media from donor HC3-26 (1 μM: 1.61 ± 0.14-fold and 10 μM: 3.48 ± 0.39-fold; Figure 4B) and donor HU1880 (1 μM: 1.24 ± 0.18-fold and 10 μM: 1.43 ± 0.09-fold, Figure 4D).

Evaluation of individual PRH effects revealed that labetalol Gluc-1 formation in SCHH was significantly increased following E2 exposure in cell lysates (Figures 4A,C) and in media (Figures 4B,D). The E2 effects in media harvested from both donors were concentration-dependent. In donor HC3-26, 10 μM P4 increased labetalol Gluc-1 formation compared to vehicle control in media (Figure 4B); however, this effect was smaller in magnitude compared to 10 μM E2 and no effect was observed in cell lysates (Figure 4A). In donor HU1880, exposure to 1 μM P4 appeared to modestly suppress Gluc-1 formation, but this effect was not observed with 10 μM P4 (Figures 4C,D). In donor HU1880, CRT appeared to increase labetalol Gluc-1 formation in cell lysates (Figure 4C), but these effects were small, not concentration-dependent, and not observed in the media harvested from donor HU1880 (Figure 4D) or in either cell lysates or media harvested from donor HC3-26 (Figures 4A,B).

In donor HC3-26, co-administration of itraconazole a UGT1A1 inhibitor, abolished the PRH CKTL and rifampin-evoked increases in labetalol Gluc-1 formation (Figures 4A,B). In addition, changes in UGT1A1 protein concentrations positively and significantly correlated with labetalol Gluc-1 formation in both SCHH cell lysate (Figure 4E; r = 0.670, *p* = 0.002) and media (Figure 4F; r = 0.763, *p* < 0.001), demonstrating that the PRH-induced increases in labetalol Gluc-1 formation were mediated by increased UGT1A1 expression.

### Effect of Pregnancy-Related Hormones on UGT2B7-Mediated Labetalol Glucuronidation in SCHH

**FIGURE 5:**
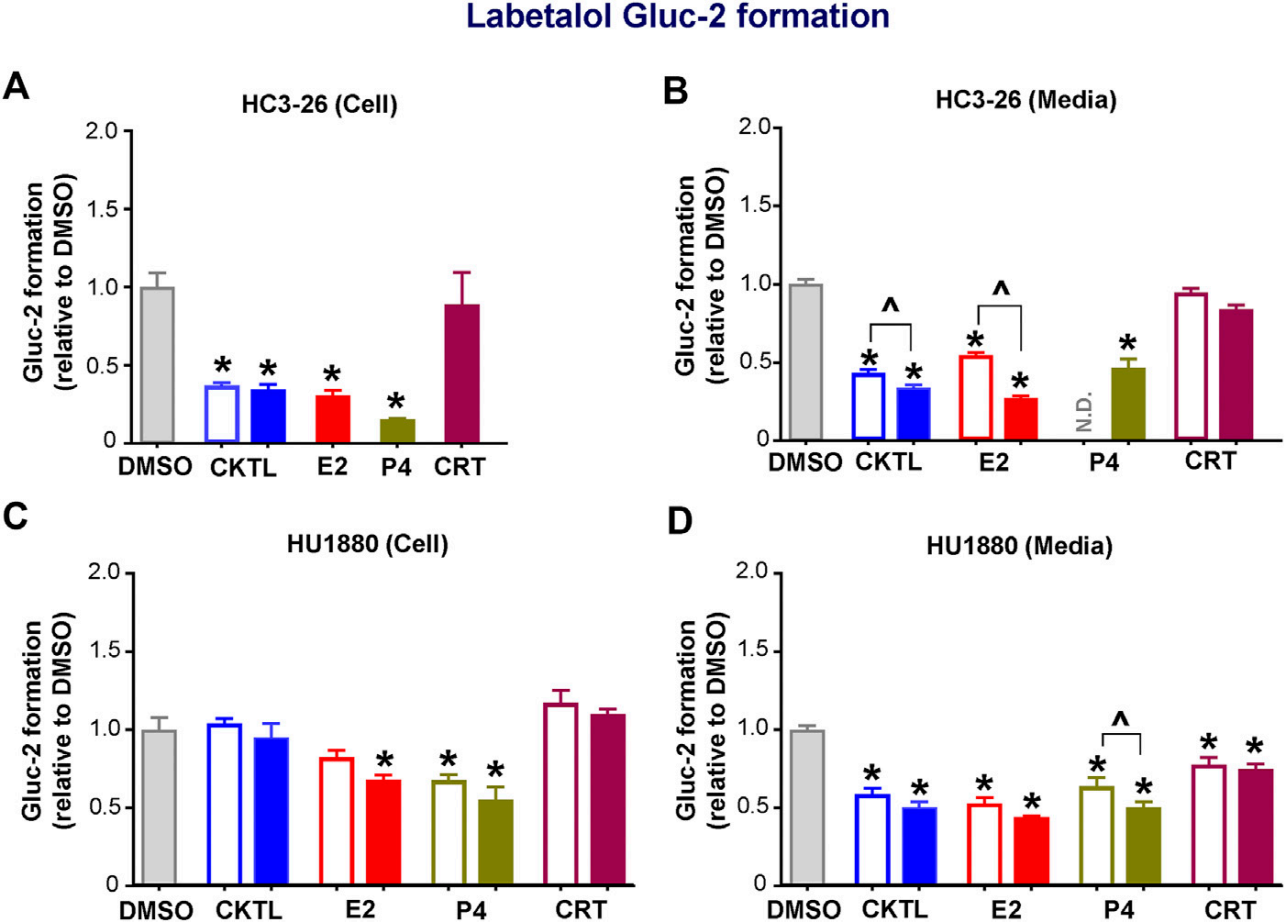
Effect of pregnancy-related hormones (PRH) on labetalol glucuronide (Gluc-2) formation in SCHH. Following 72 h of PRH exposure, SCHH from two donors (HC3-26, HU1880) were incubated with labetalol (1 mM) for 4 h. Labetalol glucuronide (Gluc-2) peak area was measured in both SCHH cell lysates **(A, C)** and SCHH media **(B, D)**, normalized to internal standard (labetalol-d_3_) peak area in each sample, and compared across treatment groups in donor HC3-26 (A: cell lysate, *n* = 3 per group; B: media, *n* = 3–6 per group) and donor HU1880 (C: cell lysate, *n* = 4 per group; D: media, *n* = 4 per group). In the donor HC3-26 experiment, cell lysates were not harvested for glucuronide measurements in the 1 µM E2, P4, or CRT groups. Data are calculated as the fold-change in Gluc-2 metabolite formation relative to vehicle control (*p < 0.05 vs. DMSO). Concentration-dependent effects were assessed (open bar: 1 μM, solid bar: 10 μM; ^*p* < 0.05 1 vs. 10 µM). N.D., experimental group not studied.

We also examined the impact of PRH on labetalol Gluc-2 formation in SCHH. Although PRH CKTL did not alter UGT2B7 protein concentrations in SCHH, the PRH CKTL significantly decreased labetalol Gluc-2 formation compared to vehicle control (Figure 5). This effect was observed in cell lysates and media harvested from hepatocyte donor HC3-26 (Figures 5A,B), and the media harvested from donor HU1880 (Figure 5D). However, a similar reduction in Gluc-2 formation was not observed in cell lysates harvested from donor HU1880 (Figure 5C). Evaluation of individual PRH effects revealed that labetalol Gluc-2 formation was decreased following exposure to E2 and P4, but not CRT, in both the cell lysates and media harvested from each donor. A modest reduction following CRT exposure was observed in media harvested from donor HU1880 (Figure 5D), but no effect was observed in the cell lysates harvested from donor HU1880 (Figure 5C) or in either cell lysates or media harvested from donor HC3-26 (Figures 5A,B).

UGT2B7 protein concentrations in SCHH membrane fractions did not positively correlate with labetalol Gluc-2 formation in media (r = −0.329, *p* = 0.182) or cell lysates (r = −0.465, *p* = 0.052), demonstrating that the PRH-evoked decreases in labetalol Gluc-2 formation were not mediated by changes in UGT2B7 expression. A follow-up experiment with recombinant human UGT2B7 enzyme demonstrated that co-administration of the PRH CKTL (1 µM) elicited a small but not statistically significant reduction in labetalol Gluc-2 formation compared to vehicle control (0.85 ± 0.05-fold vs. 1.00 ± 0.05-fold; P = 0.093; *n* = 3/group).

### Expression of UGT1A4, but not Other UGT1A Proteins, are Altered by Pregnancy-Related Hormones in SCHH

**FIGURE 6:**
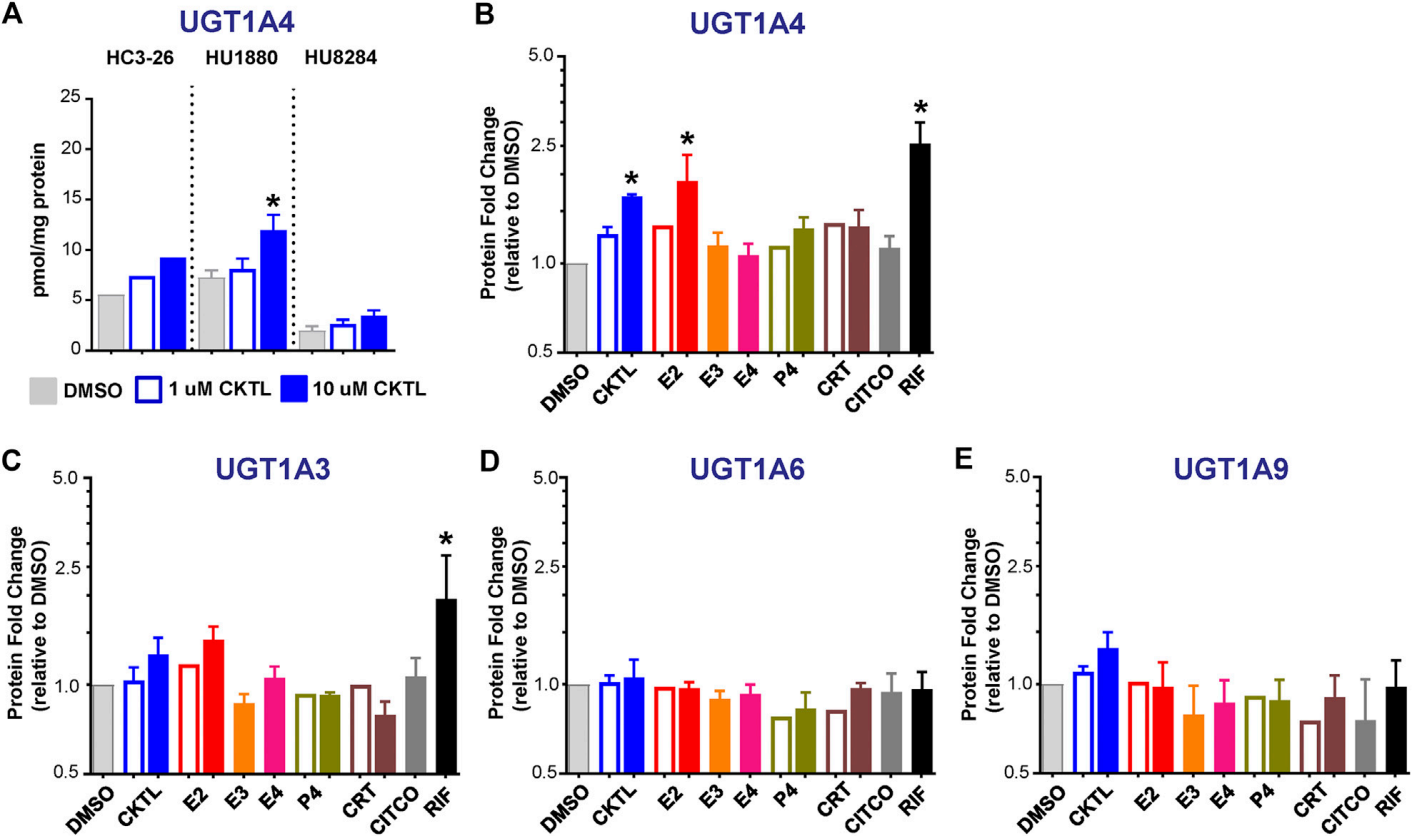
Effect of pregnancy-related hormones (PRH) on protein concentrations of UGT1A4 and other key UGT1A isoforms in SCHH. Following 72 h of PRH exposure, UGT1A4 protein concentrations were quantified by quantitative targeted absolute proteomics in SCHH membrane-associated protein fractions isolated from three donors (HC3-26, HU1880, and HU8284). **(A)** UGT1A4 absolute protein concentrations in the DMSO and PRH cocktail (CKTL) groups were compared separately in donor HC3-26 (mean: *n* = 2 per group) and donors HU1880 and HU8284 (mean ± SEM: n = 3–4 per group; *p < 0.05 vs. DMSO, ^*p* < 0.05 1 vs. 10 µM). Comparisons of PRH CKTL effects on UGT1A3, UGT1A6, and UGT1A9 concentrations within each hepatocyte donor are provided in Supplementary Figure S3. **(B)** UGT1A4, **(C)** UGT1A3, **(D)** UGT1A6, and **(E)** UGT1A9 protein concentrations were expressed relative to vehicle control (DMSO) within each hepatocyte donor, and then combined for comparison across groups. Open bars represent 1 µM CKTL (mean ± SEM: *n* = 3 donors per group) and 1 µM for the individual hormones (mean: *n* = 2 donors per group). Solid bars represent 10 µM CKTL and 10 µM for the individual hormones (mean ± SEM: *n* = 3 donors per group). *p < 0.05 vs. DMSO, ^*p* < 0.05 1 vs. 10 µM. Comparison of individual PRH effects on UGT1A4 levels within each hepatocyte donor is provided in Supplementary Figure S2C.

The impact of PRH on absolute protein concentrations of four additional UGT1A isoforms in SCHH were quantified and compared to the observed changes in UGT1A1 expression. Most notably, the PRH CKTL increased UGT1A4 protein concentrations with induction effects that varied across hepatocyte donors (Figure 6A). Analysis of the average effect across donors demonstrated that the PRH CKTL significantly increased UGT1A4 protein concentrations (Figure 6B), and this effect was similar in magnitude to the PRH-evoked induction of UGT1A1 (Figure 2C). This effect also appeared to be driven by E2, which increased UGT1A4 protein concentrations in a concentration-dependent manner and paralleled the induction effects of rifampin (Figure 6B; Supplementary Figure S2C). The PRH evoked changes in UGT1A4 protein concentrations exhibited a significant positive correlation with *UGT1A4* mRNA levels (r = 0.747, *p* < 0.001).

In contrast, UGT1A3, UGT1A6, and UGT1A9 protein concentrations were not significantly altered by PRH in SCHH. Although UGT1A3 and UGT1A9 were increased at the high CKTL concentration in donor HU1880 control (Supplementary Figures S3A,C), the average effect across donors was small in magnitude and was not significantly different compared to vehicle control (Figures 6C,E). PRH did not alter UGT1A6 protein concentrations compared to vehicle control (Figure 6D, Supplementary Figure S3B).

## Discussion

Growing evidence has demonstrated that PRH are important regulators of altered hepatic cytochrome P450 enzyme expression and function during pregnancy (Jeong, 2010; Isoherranen and Thummel, 2013; Khatri et al., 2021). However, the impact of PRH on hepatic UGT enzyme expression and function is less well understood. Notably, the impact of PRH on absolute protein concentrations of UGT1A1, UGT2B7 and other key UGT isoforms in primary SCHH, and the hepatic metabolism of clinically relevant UGT substrates commonly prescribed to pregnant individuals, had not yet been studied. In this study, we report that exposure to a PRH CKTL 1) increased UGT1A1 and UGT1A4 mRNA levels and protein concentrations in human hepatocytes to a greater degree than other UGT1A isoforms, and did not significantly alter UGT2B7 mRNA levels or protein concentrations; 2) increased hepatic UGT1A1-mediated glucuronidation of labetalol, a commonly prescribed drug to treat hypertensive disorders of pregnancy, by inducing UGT1A1 protein concentrations; and 3) decreased UGT2B7-mediated labetalol glucuronidation in human hepatocytes without decreasing UGT2B7 protein concentrations. Collectively, these findings demonstrate that PRH alter expression and function of UGT proteins in an isoform-specific manner, provide mechanistic insight into the increases in labetalol clearance observed in pregnant individuals, and provide experimental evidence supporting the hypothesis that increased UGT1A1-mediated labetalol metabolism contributes to the increased labetalol hepatic intrinsic clearance during pregnancy predicted by pharmacokinetic models (Fischer et al., 2014).

Prior studies have demonstrated that PRH increase *UGT1A1* and *UGT1A4* promoter activity in HepG2 cells by activating PXR and ERα dependent transcription, respectively; in contrast, PRH do not alter either PXR or ERα dependent *UGT2B7* promoter activation in HepG2 cells (Jeong et al., 2008; Chen et al., 2009). Consistent with these prior studies, PRH significantly increased *UGT1A1* and *UGT1A4* but not *UGT2B7* mRNA levels in primary human hepatocytes in our experiments. Because changes in DME protein concentrations more precisely correlate with metabolism changes than mRNA levels (Ohtsuki et al., 2012), we used QTAP to study the effects of PRH on absolute concentrations of multiple UGT proteins in SCHH (Khatri et al., 2019). Our results revealed that the presence and magnitude of PRH effects on UGT protein concentrations in SCHH varied across isoforms. Most notably, the PRH CKTL increased UGT1A1 and UGT1A4 protein concentrations to a greater extent compared to UGT1A3, UGT1A6, and UGT1A9, and did not alter UGT2B7 protein concentrations. The PRH-evoked increase in *UGT1A1* mRNA levels and UGT1A1 protein concentrations in our SCHH experiments was in alignment with prior studies demonstrating PRH induction of *UGT1A1* mRNA levels in hepatocytes isolated from hUGT1 mice and higher liver expression of UGT1A1 and Ugt1a1 in pregnant hUGT1 and wild-type mice, respectively, compared to non-pregnant controls (Chen et al., 2012; Liao et al., 2018).

The clearance of labetalol, a UGT1A1 and UGT2B7 substrate commonly prescribed for hypertensive disorders of pregnancy, has been reported to increase approximately 1.5- to 2-fold during pregnancy, leading to lower labetalol plasma concentrations, higher risk of treatment failures, and higher doses or more frequent dosing to lower blood pressure; however, substantial inter-patient variability in these changes exists (Rogers et al., 1990; Saotome et al., 1993; Kirsten et al., 1998; Hardman et al., 2005; Fischer et al., 2014; Clark et al., 2015). A population pharmacokinetic analysis of gestational changes in labetalol pharmacokinetics concluded that the observed increase in labetalol oral clearance during pregnancy was most likely mediated by an increase in hepatic intrinsic clearance (Fischer et al., 2014). Our experiments in SCHH demonstrated that PRH CKTL significantly increased labetalol metabolism to the UGT1A1-derived glucuronide metabolite in a concentration-dependent manner (1.3-fold–3.5-fold across hepatocyte donors), which positively correlated with PRH-induced increases in UGT1A1 protein concentrations. In contrast, PRH CKTL did not increase UGT2B7 protein concentrations or UGT2B7-mediated labetalol glucuronidation in SCHH. While we cannot draw a direct quantitative comparison between our *in vitro* labetalol metabolism experiments and increased labetalol clearance in pregnant individuals, these results provide experimental evidence suggesting that induction of hepatic UGT1A1, not UGT2B7, protein concentrations underlies the increased hepatic labetalol metabolic clearance during pregnancy predicted by pharmacokinetic models (Fischer et al., 2014; Clark et al., 2015). Although future *in vivo* studies are needed to validate this hypothesis, and investigate other potential mechanisms that could increase labetalol oral clearance during pregnancy (e.g., intestinal UGT metabolism, hepatic transport), these effects are consistent with prior studies demonstrating a parallel *in vitro* induction of *UGT1A4* expression and lamotrigine glucuronidation (a UGT1A4 substrate) by E2 in HepG2 cells (Chen et al., 2009) and an *in vivo* increase in lamotrigine glucuronide metabolite formation, oral clearance, and dose requirements in pregnant individuals (Ohman et al., 2008a; Ohman et al., 2008b; Pennell et al., 2020).

Induction of UGT1A1 expression and metabolism by the PRH CKTL in SCHH was concentration-dependent, driven by E2, and mirrored the induction effects of the PXR activator rifampin. However, contrary to our expectation, UGT1A1 expression and metabolism were minimally impacted by P4 and CRT. *UGT1A1* mRNA levels are significantly increased by E2, P4, and corticosterone in hepatocytes isolated from hUGT1 mice (Chen et al., 2012). Moreover, P4 was a more potent inducer of *UGT1A1* promoter activation and mRNA levels compared to E2 in HepG2 cells transfected with pcDNA3-PXR (Jeong et al., 2008). The differences in individual hormone effects between our experiments in SCHH and these prior studies in hepatocytes isolated from hUGT1 mice and pcDNA3-PXR transfected HepG2 cells may be due to differences in experimental models. For instance, it is not known whether differences in basal UGT1A1 expression exist across models. Furthermore, these prior studies used hepatocyte culture media that was deplete of dexamethasone. Thus, the lower magnitude of the P4 and CRT induction effect in our SCHH experiments could be due to presence of dexamethasone or other hormones in the commercially obtained hepatocyte media. The observed PRH-evoked increase in UGT1A4 expression in our experiments was also driven by E2. This observation was consistent with previous studies reporting E2-mediated increases in *UGT1A4* promoter activation and mRNA levels and lamotrigine glucuronidation in pcDNA3-ERα transfected HepG2 cells (Chen et al., 2009).

Our results also illustrated that the presence and magnitude of PRH effects on UGT1A protein concentrations varied across the tested hepatocyte donors. For example, the PRH CKTL increased UGT1A3 and UGT1A9 protein concentrations in donor HU1880; however, changes were not observed in donor HC3-26 or HU8284. In addition, hepatocyte donor-to-donor variation in the degree of UGT1A1 and UGT1A4 induction was observed. Hepatocyte donor differences in DME induction by prototypical inducers and PRH has been reported previously (Schaefer et al., 2012; Choi et al., 2013; MacLean et al., 2017; Khatri et al., 2021); however, the mechanisms are unclear and require further study. Although this study is the first to quantify PRH effects on absolute UGT protein concentrations in human hepatocytes, we acknowledge our study remains limited by the relatively small number of hepatocyte donors studied. Accordingly, while PRH CKTL did not alter UGT1A3, UGT1A6, UGT1A9, or UGT2B7 protein concentrations in these donors, effects may be present in other donors. Furthermore, prior studies have demonstrated that PRH combinations can elicit additive and synergistic effects on the induction of *CYP3A4* mRNA levels and midazolam metabolism (Papageorgiou et al., 2013). In addition, other PRH such as placental growth hormone and placental lactogen have been reported to alter cytochrome P450 mRNA levels (Lee et al., 2014), but their effects on UGT expression remains unknown. Future experiments that evaluate a larger number of hepatocyte donors and PRH combinations are necessary to define the magnitude and variability of these effects more precisely, discern additive and synergistic effects between hormones, and elucidate the underlying mechanisms. Studies evaluating PRH effects on absolute protein concentrations and function of other key UGT isoforms, as well as carboxylesterase and sulfotransferase enzymes and drug uptake and efflux transporters, in human hepatocytes are also needed. Our study provides a foundation to design and interpret these experiments, which will be more feasible as more sensitive sample processing and QTAP methods allow higher throughput screening with fewer hepatocytes (Qasem et al., 2021).

Our observation that UGT2B7-derived labetalol glucuronide metabolite formation was decreased in SCHH following PRH exposure was unexpected. Given the absence of PRH-evoked decreases in UGT2B7 expression in SCHH, the observed reduction of labetalol Gluc-2 formation following PRH CKTL treatment was not mediated by suppression of UGT2B7 protein expression. It is well-established that E2, E3, E4, and CRT undergo glucuronidation, and that rates of UGT2B7-mediated glucuronidation of E2 and E3 are very high (Gall et al., 1999; Visser et al., 2008; Wang et al., 2018). Moreover, while P4 does not directly undergo glucuronidation, the P4 metabolites pregnanediol and pregnanetriol are metabolized to their respective glucuronides (Meng et al., 1996). Although the underlying mechanisms and relevance to labetalol metabolism *in vivo* remain unclear, these data suggest that certain PRH and/or their metabolites could potentially interfere with UGT2B7-mediated labetalol glucuronidation in cultured human hepatocytes. Future studies that quantify and compare PRH and metabolite effects on UGT2B7 activity, and elucidate inhibition mechanisms, are warranted.

Given the contributions of hepatic glucuronidation to labetalol clearance *in vivo* and both UGT1A1 and UGT2B7 to labetalol glucuronidation *in vitro* (Martin et al., 1976; Yeleswaram et al., 1993; Jeong et al., 2008), and our observations that PRH can alter UGT1A1-and UGT2B7-mediated glucuronidation of labetalol in SCHH via distinct mechanisms, inter-individual differences in these effects could potentially contribute to the substantial inter-patient variability in pregnancy-induced changes in labetalol pharmacokinetics reported in the literature (Rogers et al., 1990; Saotome et al., 1993; Kirsten et al., 1998; Hardman et al., 2005; Fischer et al., 2014; Clark et al., 2015). It is important to note, however, that the relative contribution of UGT1A1-and UGT2B7-mediated glucuronidation to labetalol clearance *in vivo* has not been reported in the literature. This gap in evidence may be due to the lack of labetalol glucuronide analytical standards and quantitative methods that can measure absolute concentrations of distinct labetalol glucuronides, which are needed to quantify and compare labetalol formation clearance to its UGT1A1 and UGT2B7-derived metabolites. Future human pharmacokinetic studies that quantify and compare the metabolic clearance of UGT1A1-and UGT2B7-derived labetalol glucuronides in non-pregnant and pregnant individuals are needed to elucidate the relative contribution of these hepatic clearance pathways to labetalol pharmacokinetic changes during pregnancy. Our *in vitro* experiments in SCHH lay a foundation for these future studies.

## Conclusion

To our knowledge, this is the first study in primary human hepatocytes to evaluate the impact of PRH on UGT mRNA levels and protein concentrations, and the glucuronidation of labetalol, a UGT1A1 and UGT2B7 substrate commonly prescribed to treat hypertensive disorders of pregnancy. We report that PRH CKTL increased mRNA and protein levels of UGT1A1 and UGT1A4 to a greater extent than other UGT1A isoforms, and increased labetalol metabolism to its UGT1A1-derived glucuronide metabolite by inducing UGT1A1 expression. In contrast, PRH CKTL decreased UGT2B7-mediated labetalol glucuronidation in SCHH without altering UGT2B7 protein expression. Our data demonstrate that PRH alter UGT expression and function in human hepatocytes in an isoform-specific manner, and provide experimental evidence supporting the hypothesis that increased UGT1A1-mediated hepatic labetalol metabolism contributes to the increased labetalol clearance observed during pregnancy in humans (Fischer et al., 2014; Clark et al., 2015). These results provide a foundation for future experiments that investigate the effects of PRH on UGT-catalyzed metabolism of other drugs prescribed during pregnancy, and offer the potential to inform pharmacokinetic models and more precise labetalol dosing recommendations in pregnant patients.

## Data Availability

The raw data supporting the conclusion of this article will be made available by the authors, without undue reservation.

## Abbreviations

CAR, constitutive androstane receptor; CITCO, (6-4-Chlorophenyl) Imidazo [2,1-b][1,3] Thiazole-5-Carbaldehyde o-(3,4-dichlorobenzyl) Oxime; CKTL, cocktail; CRT, cortisol; DMSO, dimethyl sulfoxide; E2, estradiol; E4, estetrol; E3, estriol; ERα, estrogen receptor alpha; GAPDH, glyceraldehyde 3-phosphate dehydrogenase; P4, progesterone; PBPK, physiologically based pharmacokinetic; PRH, pregnancy-related hormones; PXR, pregnane X receptor; QTAP, quantitative targeted absolute proteomics; SCHH, sandwich-cultured human hepatocytes; UDPGA, uridine-diphosphate-glucuronic acid; UGT, Uridine 5′-diphospho-glucuronosyltransferase.
