# Supplementary material for: Pregnancy-Related Hormones Increase UGT1A1-Mediated Labetalol Metabolism in Human Hepatocytes
Source: Front Pharmacol. 2021 Apr 15;12:655320. doi: 10.3389/fphar.2021.655320 (PMC8115026; doi:10.3389/fphar.2021.655320)
Supplement: Supplementary file 1 [file Table1.PDF]

Supplemental Table 1. Characteristics of the female human hepatocyte donors.

**Vendor**	**Lot**	**Age** (years)	**Gender**	**Race**	**Serology**	**Drug History**	**Cause of death**
Fisher Scientific	HU1880	34	F	Cauc	-	Lyrica, Vitamin B	Not reported
Xenotech	HC3-26 [a]	43	F	Cauc	-	Not reported	Cerebrovascular accident
Fisher Scientific	HU8284	46	F	Cauc	-	None	Self inflicted gunshot wound
Xenotech	HC5-40 [b]	49	F	Cauc	CMV+	Not reported	Anoxia

[a] Hepatocytes from this lot were Transporter Certified™ (BioIVT, Durham, NC)
[b] Hepatocytes from this lot exhibited suboptimal cell viability in culture during the mRNA induction experiment, and thus were not used for the subsequent quantitative proteomics and labetalol metabolism experiments.
