# Supplementary material for: Pregnancy-Related Hormones Increase UGT1A1-Mediated Labetalol Metabolism in Human Hepatocytes
Source: Front Pharmacol. 2021 Apr 15;12:655320. doi: 10.3389/fphar.2021.655320 (PMC8115026; doi:10.3389/fphar.2021.655320)
Supplement: Supplementary file 2 [file Table2.PDF]

Supplemental Table 2. **Human proteotypic tryptic peptides used to report UGT concentrations in SCHHs, and the MRMs acquired.**

UGT isoform	Peptide Sequence	MRM1 (product ion) (mass spec specific)	MRM2 (product ion) (mass spec specific)
UGT1A1	$D_{70}GA\mathbf{F}YTLK_{77}$	462.75/681.39 (y5)	462.75/524.31 (y4)
UGT1A3	$Y_{164}LSIPTVFFL\mathbf{R}_{174}$	683.39/1089.63 (y9)	683.39/889.52 (y7)
UGT1A4	$Y_{164}LSIPAVFFW\mathbf{R}_{174}$	704.89/1132.61 (y9)	704.89/932.51 (y7)
UGT1A6	$D_{44}IVEVLSD\mathbf{R}_{52}$	528.28/728.38 (y6)	528.28/599.34 (y5)
UGT1A9	$G_{171}ILCHYLEEGAQCPAPLSYVP\mathbf{R}_{192}$	847.41/1009.56 (y9)	847.41/841.48 (y7)
UGT2B7	$A_{253}DVWLI\mathbf{R}_{259}$	441.76/597.39 (y4)	441.76/696.43 (y5)

MRMs are roughly in order of highest intensity.

Amino acids in bold are ^{13}C and ^{15}N heavy labeled.

Mass differences between labeled (shown) and unlabeled (not shown) R and F is 10 (m/z differences also depend on charge state).
