# Supplementary material for: Pregnancy-Related Hormones Increase UGT1A1-Mediated Labetalol Metabolism in Human Hepatocytes
Source: Front Pharmacol. 2021 Apr 15;12:655320. doi: 10.3389/fphar.2021.655320 (PMC8115026; doi:10.3389/fphar.2021.655320)
Supplement: Supplementary file 3 [file Image2.PDF]

Supplemental Figure 2.

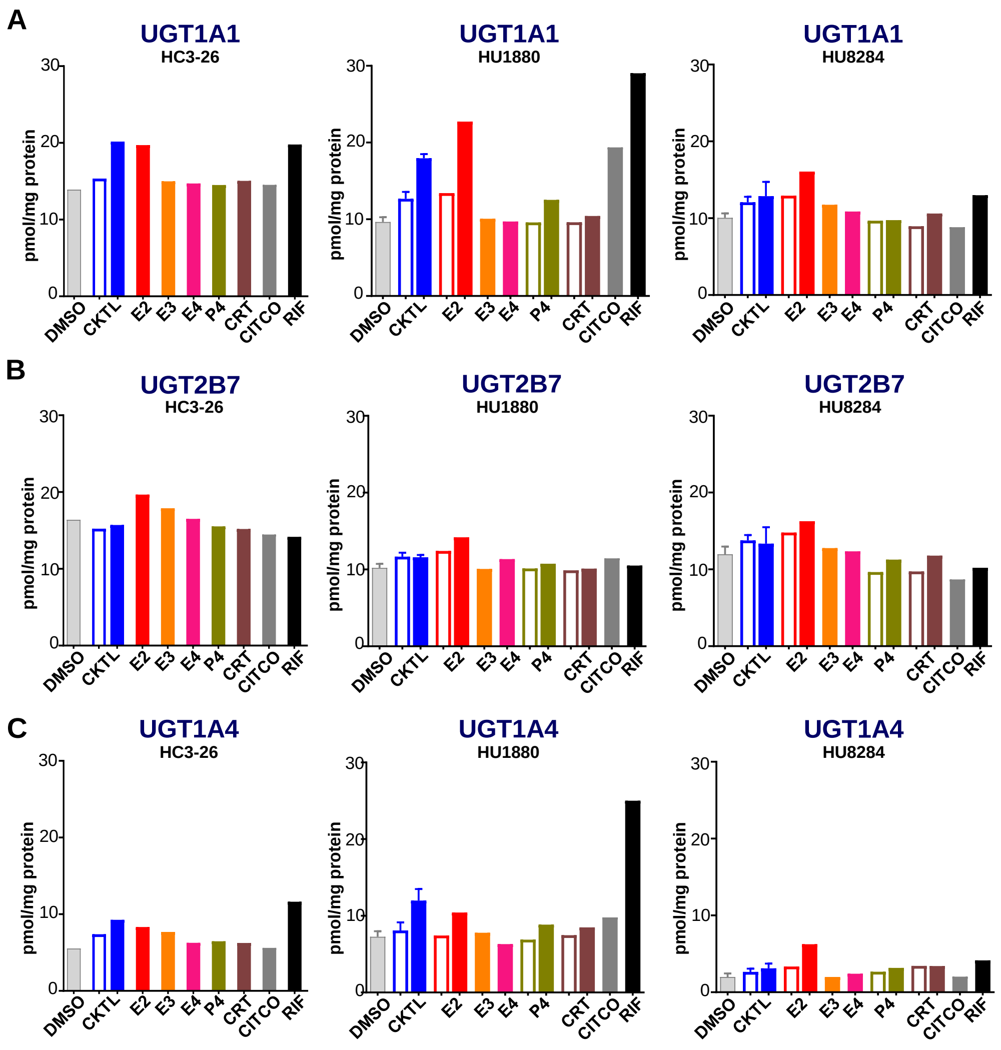

Supplemental Figure 2. Effect of pregnancy-related hormones on protein concentrations of UGT1A1, UGT2B7 and UGT1A4 in SCHH by hepatocyte donor. Following 72 h of hormone exposure, (A) UGT1A1, (B) UGT2B7, and (C) UGT1A4 protein concentrations were quantified by quantitative targeted absolute proteomics in SCHH membrane-associated protein fractions isolated from three donors (HU1880, HC3-26, HU8284). Absolute protein concentrations in the individual hormone (E2, E3, E4, P4, CRT) and control (CITCO, RIF) treatment groups were evaluated in each donor (mean: n=2/group). Concentration-dependent effects (open bar: 1 µM, solid bar: 10 µM) were evaluated for E2, P4 and CRT in donors HU1880 and HU8284 only. Protein concentrations in the DMSO and hormone cocktail (CKTL) groups in donors HU1880 and HU8284 (mean ± SEM: n=3-4/group) and donor HC3-26 (mean: n=2/group), which are also presented in Figure 2 (UGT1A1, UGT2B7) and Figure 6 (UGT1A4), are included for comparison.
