# Supplementary material for: Pregnancy-Related Hormones Increase UGT1A1-Mediated Labetalol Metabolism in Human Hepatocytes
Source: Front Pharmacol. 2021 Apr 15;12:655320. doi: 10.3389/fphar.2021.655320 (PMC8115026; doi:10.3389/fphar.2021.655320)
Supplement: Supplementary file 4 [file Image3.PDF]

Supplemental Figure 3.

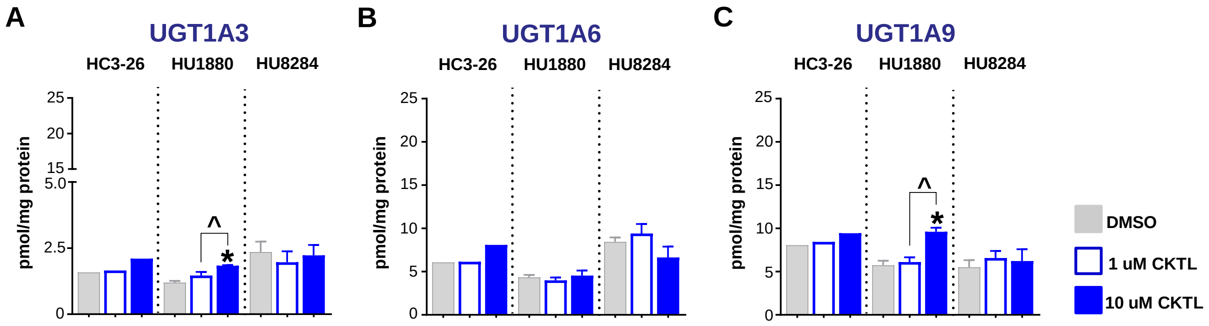

Supplemental Figure 3. Effect of the pregnancy-related hormone cocktail on protein concentrations of UGT1A3, UGT1A6, and UGT1A9 in SCHH by hepatocyte donor. Following 72 h of hormone exposure, UGT1A3, UGT1A6, and UGT1A9 protein concentrations were quantified by quantitative targeted absolute proteomics in SCHH membrane-associated proteins isolated from three donors (HC3-26, HU1880, and HU8284). (A) UGT1A3, (B) UGT1A6, and (C) UGT1A9 absolute protein concentrations in the DMSO and hormone cocktail (CKTL) groups were compared separately in donor HC3-26 (mean: n=2/group) and donors HU1880 and HU8284 (mean ± SEM: n=3-4/group; *P<0.05 vs. DMSO, ^P<0.05 1 vs. 10 µM). Open bars represent 1 µM CKTL. Solid blue bars represent 10 µM CKTL.
