# Supplementary material for: Pregnancy-Related Hormones Increase UGT1A1-Mediated Labetalol Metabolism in Human Hepatocytes
Source: Front Pharmacol. 2021 Apr 15;12:655320. doi: 10.3389/fphar.2021.655320 (PMC8115026; doi:10.3389/fphar.2021.655320)
Supplement: Supplementary file 5 [file Image1.PDF]

Supplemental Figure 1.

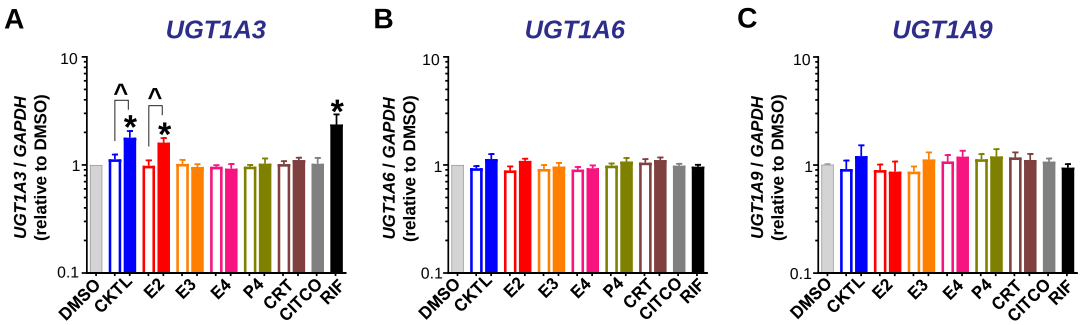

Supplemental Figure 1. Effect of pregnancy-related hormones on *UGT1A3*, *UGT1A6*, and *UGT1A9* mRNA levels in SCHH. Human hepatocytes from four female donors (HU1880, HC3-26, HU8284, and HC5-40) were exposed to hormones (E2, E3, E4, P4, CRT), either individually or in combination as a cocktail [CKTL] of all hormones, or controls (DMSO, CITCO, Rifampin [RIF]) for 72 h (n=2/group within each donor). (A) *UGT1A3*, (B) *UGT1A6*, and (C) *UGT1A9* mRNA levels were quantified, normalized to GAPDH, expressed relative to the vehicle control (DMSO) within each donor, and then combined for comparison across experimental groups (n=4 donors/group; mean ± SEM; *p<0.05 vs. DMSO). Concentration-dependent effects were evaluated (open bar: 1 µM, solid bar: 10 µM; ^p<0.05 1 vs. 10 µM).
